# Endometrial glands as a source of nutrients, growth factors and cytokines during the first trimester of human pregnancy: A morphological and immunohistochemical study

**DOI:** 10.1186/1477-7827-2-58

**Published:** 2004-07-20

**Authors:** Joanne Hempstock, Tereza Cindrova-Davies, Eric Jauniaux, Graham J Burton

**Affiliations:** 1 Department of Anatomy, University of Cambridge, Cambridge, UK; 2 Academic Department of Obstetrics and Gynaecology, Royal Free and University College, London, UK

## Abstract

**Background:**

The maternal circulation to the human placenta is not fully established until 10–12 weeks of pregnancy. During the first trimester the intervillous space is filled by a clear fluid, in part derived from secretions from the endometrial glands via openings in the basal plate. The aim was to determine the activity of the glands throughout the first trimester, and to identify components of the secretions.

**Methods:**

Samples of human decidua basalis from 5–14 weeks gestational age were examined by transmission electron microscopy and immunohistochemically. An archival collection of placenta-in-situ samples was also reviewed.

**Results:**

The thickness of the endometrium beneath the implantation site reduced from approximately 5 mm at 6 weeks to 1 mm at 14 weeks of gestation. The glandular epithelium also transformed from tall columnar cells, packed with secretory organelles, to a low cuboidal layer over this period. The lumens of the glands were always filled with precipitated secretions, and communications with the intervillous space could be traced until at least 10 weeks. The glandular epithelium reacted strongly for leukaemia inhibitory factor, vascular endothelial growth factor, epidermal growth factor, transforming growth factor beta, alpha tocopherol transfer protein, MUC-1 and glycodelin, and weakly for lactoferrin. As gestation advanced uterine natural killer cells became closely approximated to the basal surface of the epithelium. These cells were also immunopositive for epidermal growth factor.

**Conclusions:**

Morphologically the endometrial glands are best developed and most active during early human pregnancy. The glands gradually regress over the first trimester, but still communicate with the intervillous space until at least 10 weeks. Hence, they could provide an important source of nutrients, growth factors and cytokines for the feto-placental unit. The endometrium may therefore play a greater role in regulating placental growth and differentiation post-implantation than previously appreciated.

## Background

The realisation that the maternal circulation to the human placenta is extremely limited prior to 10–12 weeks of pregnancy prompted us to investigate other potential sources of fetal nutrition during the first trimester [1-4]. During the evolution of ovoviviparity and viviparity secretions from the uterus became an increasingly important supplement to the yolk contained within the embryo's yolk sac [5]. In chondrichthyan fishes they represent an important source of nutrients, even in those species that do not possess a placenta [6]. Amongst eutherian mammals the uptake of secretions derived from the endometrial glands by the trophoblast continues to provide an important pathway for nutritional exchange in the earliest stages of pregnancy, before the placenta is established. These secretions contain a complex array of carbohydrates, proteins and lipids, and have been referred to variously as uterine milk or histiotroph [5]. They are particularly significant in ruminants and equids where there is a relatively long interval between the arrival of the conceptus within the uterine cavity and the establishment of placentation. In some species, such as the pig and mouse, they represent a parallel pathway for the exchange of large proteins throughout most of pregnancy [7,8].

More recently, it has been appreciated that the secretions may perform wider functions beyond the simple provision of nutrients. Some components, such as glycodelin, have potent immunosuppresive properties [9], while others, such as leukaemia inhibitory factor (LIF) and MUC-1, play key roles in regulating implantation [10,11]. Histiotroph may therefore modulate materno-fetal interactions and regulate diverse aspects of placental development. Its importance during the preimplantation period has been powerfully demonstrated in the sheep, where suppression of endometrial gland development leads to failure of the conceptus to survive and develop [12]. Equally, in the horse increased expression of epidermal growth factor (EGF) in the endometrial glands correlates closely both temporally and spatially with cell proliferation in the overlying fetal membranes [13].

In the human histiotrophic nutrition has always been considered to be of little importance for two principal reasons. Firstly, the invasive form of implantation displayed by the human blastocyst removes it from the uterine lumen, and hence it was believed the uterine secretions, by day 7–10 post-fertilisation. Secondly, the contemporaneous appearance of maternal erythrocytes within the lacunar spaces of the syncytiotrophoblastic mantle has been widely interpreted as evidence of early onset of the maternal circulation, and hence haemotrophic exchange [14,15]. However, there is now a substantial body of evidence from a variety of techniques indicating that an effective maternal circulation is not established until the start of the second trimester [1,3,16,17]. Indeed, the human placenta cannot be considered haemochorial prior to this time for the intervillous space is filled with a clear fluid only [16]. Initially it was considered this fluid was derived as a plasma filtrate percolating through the trophoblastic plugs occluding the tips of the spiral arteries. However, we recently demonstrated that the uterine glands deliver secretions into the intervillous space until at least 10 weeks of gestation, suggesting that they may at least contribute to formation of the fluid [18]. This raises the possibility that they may play greater roles during early pregnancy than previously anticipated, equivalent to those in other species.

The aim of this study was therefore to examine the secretory activity of the endometrial glands within the decidua basalis both morphologically and immunohistochemically over the first trimester in order to assess their potential contribution to fetal nutrition and placental development.

## Methods

Samples of decidua basalis were obtained with informed written consent from patients undergoing surgical termination of normal pregnancies at University College Hospital, London. The study had been approved by the University College London Hospitals Committee on the Ethics of Human Research. Samples were obtained under ultrasound guidance, and were available from 30 cases ranging in gestational age from 5 weeks to 14 weeks (median age 9 weeks). Gestational age was estimated from the crown rump length of the fetus. Immediately after removal the tissues were fixed by immersion for 2 hours in either 4% paraformaldehyde in 0.1 M PIPES buffer for immunohistochemistry or 2% glutaraldehyde for electron microscopy, or frozen in OCT medium.

### Colourimetric immunohistochemistry

Paraformaldehyde-fixed tissues were embedded in paraffin wax and sectioned at 5 μm. After blocking of endogenous peroxidases by incubation with 1% H_2_O_2 _for 30 min, the sections were incubated with non-immune serum for 20 min. The primary antibodies (Table 1) were applied for 3 hrs at room temperature, and binding was detected using Vectastain Elite ABC kits (Vector Laboratories) and SigmaFast DAB (Sigma), according to the manufacturers' instructions. Sections were then lightly counterstained with haematoxylin. When necessary antigen retrieval was performed prior to blocking using 0.01 M sodium citrate buffer at pH6.0 in a pressure cooker for 3 min. Negative controls were performed by omission of the primary antibody.

**Table 1:** Primary antibodies used for immunohistochemistry.

Antigen	Species	Type	Dilution	Retrieval	Supplier
Alpha tocopherol transfer protein	Rabbit	Polyclonal	1:300	Yes	Dr D Kaempf-Rotzoll
Cathepsin D	Rabbit	Polyclonal	1:200	No (frozen)	Biogenesis
CD56	Mouse	Monoclonal	1:100	Yes	Zymed
CD68	Mouse	Monoclonal	1:100	Yes	Dako
Cytokeratin 7	Mouse	Monoclonal	1:100	No	Dako
Epidermal growth factor	Rabbit	Polyclonal	1:50	No	Autogen Bioclear
Glycodelin	Mouse	Monoclonal	1:10	No (frozen)	Prof. M Seppälä
Human placental lactogen	Rabbit	Polyclonal	1:200	Yes	Dako
Lactoferrin	Rabbit	Polyclonal	1:200	No	Dako
Leukaemia inhibitory factor	Goat	Polyclonal	1:200	No	Santa Cruz
R5Mucin-1	Mouse	Monoclonal	1:100	Yes	Abcam
Transforming growth factor β_3_	Rabbit	Polyclonal	1:300	Yes	Santa Cruz
Vascular endothelial growth factor	Goat	Polyclonal	1:400	No	Santa Cruz

### Fluorescent dual-labelling immunohistochemistry

Paraformaldehyde-fixed samples of decidua basalis were embedded in paraffin wax, and sectioned at 5 μm. After rehydration sections were subjected to antigen retrieval by proteinase K (20 μg/ml for 30 min), permeabilised in TBS containing Triton X-100 (0.1%) and Tween 20 (0.1%) (TBS-TT) for 30–60 min and blocked in 5 % goat serum for 30 min at room temperature. A mixture of a rabbit polyclonal and a mouse monoclonal antibody diluted in TBS-TT was applied, and sections were incubated overnight at 4°C. Negative control sections were left at the blocking stage and were not covered with primary antibodies. After three 10-minute washes in TBS-TT, sections were incubated for 1 hr at room temperature with a mixture of fluorescent secondary antibodies, containing goat anti-rabbit Alexa 488 and goat anti-mouse Alexa 568 (both used 1/200; Molecular Probes) in TBS-TT. Sections were washed in TBS-TT as before and then twice in distilled water for 5 min and subsequently mounted in Vectashield mounting medium containing DAPI (Vector, UK).

Frozen sections were used for dual labelling with anti-cathepsin D (rabbit) and glycodelin (mouse). Samples were frozen in cryoembedding medium. Sections (10–12 μm) were cut on a Reichert cryomicrotome, air-dried, fixed briefly in cold methanol/acetone (at -20°C) and permeabilised in TBS-TT for 30–60 min. All subsequent immunolabelling steps were carried out as with the paraffin embedded sections. Images were captured using a Leica confocal microscope (LeicaTCS-NT, Leica Instruments GmbH, Germany).

### Electron microscopy

Glutaraldehyde fixed tissues were secondary fixed in 1% osmium tetroxide for 1 hour, and embedded in Araldite epoxy resin. Semi-thin sections (1 μm) were stained with methylene blue, whereas ultra-thin sections (50 nm) were counterstained with uranyl acetate followed by lead citrate and viewed using a Philips CM100 microscope (Eindhoven, The Netherlands).

### Archival histological material

The Boyd Collection housed within the Department of Anatomy, University of Cambridge, contains a number of placenta-in-situ specimens. Only those with no recorded history of pathology were reviewed, and twelve specimens met this criterion. The gestational age (from the last menstrual period) was estimated from the recorded crown-rump length [19], and ranged from 43 to 130 days. For each specimen the thickness of the endometrium was measured from the junction of the endometrium with the cytotrophoblastic shell perpendicularly to the border of the endometrium with the myometrium at a minimum of 50 randomly selected points spread over at least 5 slides using the VIDS system (Synoptics, Cambridge).

### Statistical analyses

All analyses were performed using Statview (SAS Institute Inc., Cary, USA).

## Results

### Endometrial histology

**Figure 1:**
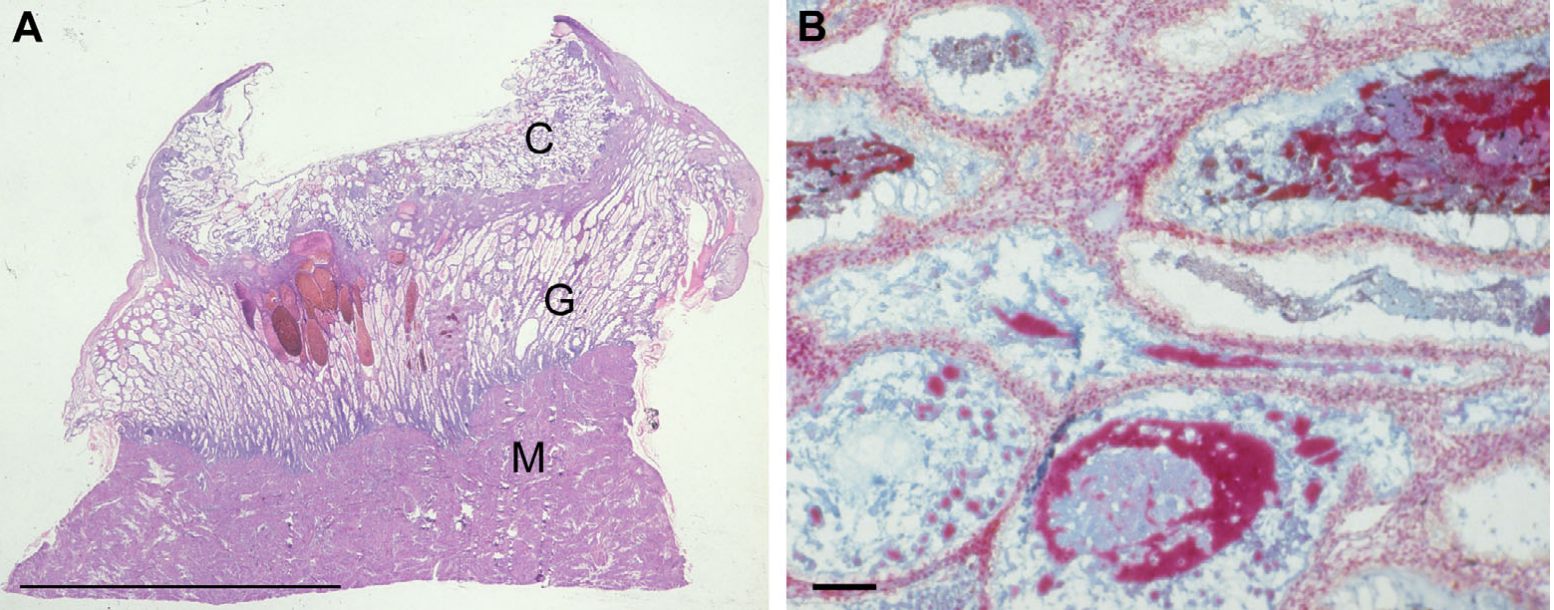
A) In the earliest specimen available, H710, the conceptus (C) can be seen embedded in the superficial endometrium overlying well-developed endometrial glands (G). M, myometrium. (Haematoxylin and eosin) Scale bar = 1.0 cm. B) The secretions within the lumens of the glands are heterogenous, being a mixture of carbohydrate-rich flocculent material (blue) and what appear to be lipid droplets (red). (Alcian blue and Neutral red) Scale bar = 100 μm.

**Figure 2:**
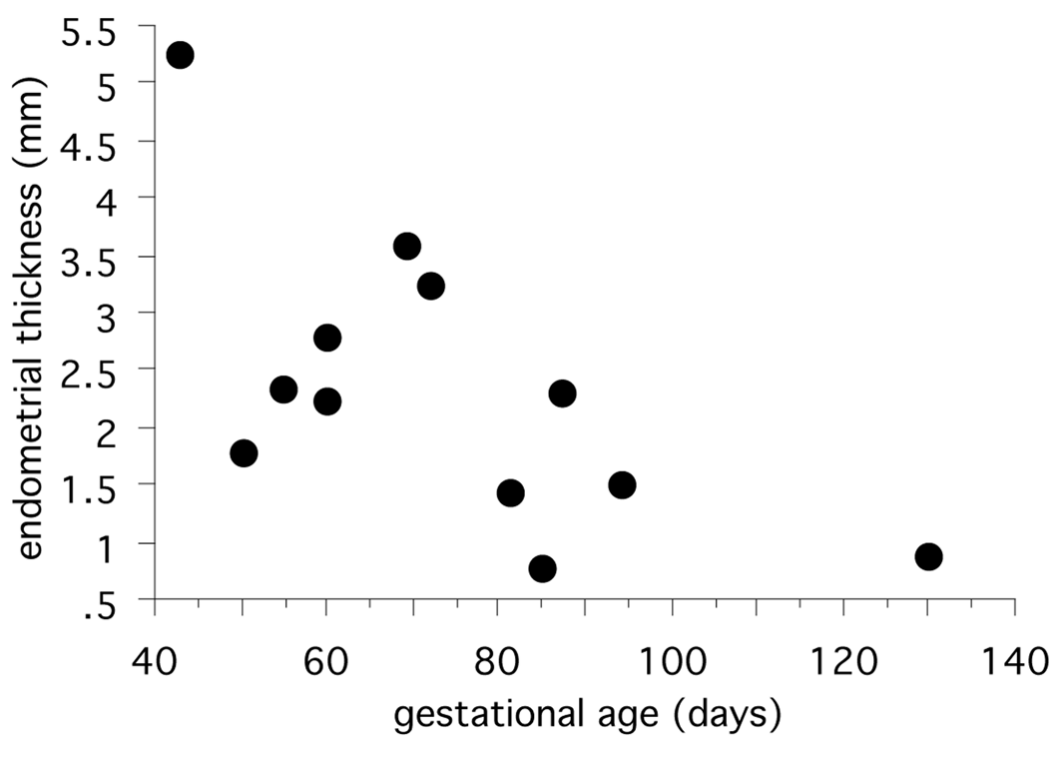
Scattergram showing the relationship between endometrial thickness and gestational age.

**Figure 3:**
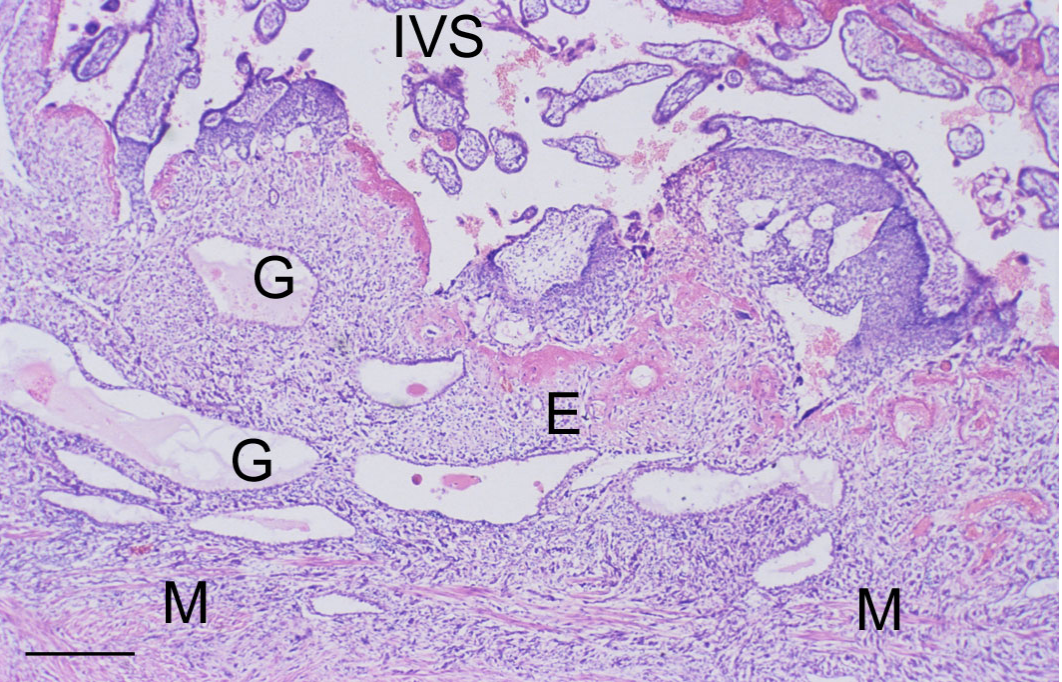
Placenta-in-situ specimen (H1094) of 13.5 weeks gestational age showing the reduction in thickness of the endometrium (E) at this stage of pregnancy. The glands (G) have a more regular outline, but still contain precipitated secretions within their lumens. M, myometrium; IVS, intervillous space. (Haematoxylin and eosin) Scale bar = 1.0 mm.

In the earliest specimen available, H710 estimated to be of 43 days menstrual age, the conceptus was embedded within the superficial layer of a highly secretory endometrium (Figure 1A). The uterine glands displayed the sawtooth appearance characteristic of the late secretory phase of the menstrual cycle, and were filled with copius secretions. These were heterogenous in nature, comprising a carbohydrate-rich flocculent material in which were interspersed numerous smooth round droplets resembling lipid (Figure 1B). The cytotrophoblastic shell was well developed, and formed a smooth interface with the endometrium. As gestational age advanced the thickness of the decidua basalis reduced dramatically from over 5 mm at 6 weeks to approximately 1 mm at 14 weeks (Figure 2). Although there was considerable variation between samples a statistically significant negative correlation existed between the two parameters (*r *= -0.644, *P *= 0.0216). As gestation advanced there was also increasing variability in the thickness of the endometrium across the placental bed, reflecting the formation of placental septa. The profiles of the glands became smoother and more regular, but they still contained precipitated secretions (Figure 3). Communications with the intervillous space could be traced until at least 10 weeks gestational age.

### Glandular epithelium

**Figure 4:**
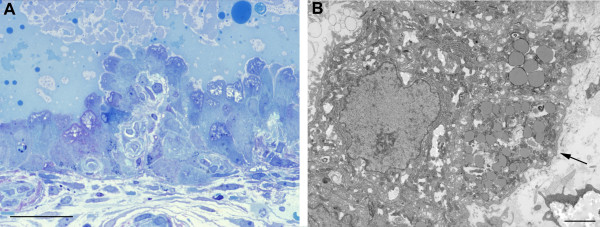
A) Photomicrograph of a 1 μm resin section of 6 week decidua illustrating the columnar epithelium of the glands, their large apical projections and the heterogeneous nature of the secretions. (Methylene blue) Scale bar = 10 μm. B) At the ultrastructural level it can be seen that the cells possess large quantities of mitochondria and endoplasmic reticulum, and lipid droplets are abundant in the basal region. The cells are attached to a well-formed basal lamina (arrowed). Scale bar = 2 μm.

In the early specimens the epithelial cells displayed a tall columnar morphology, often with large apical projections extending into the glandular lumen (Figure 4A). This was confirmed at the ultrastructural level, at which it could be seen that the apical membrane bounding these projections displayed only scanty short microvilli. Tight junctions were present at the base of the projections, linking the cells. Within the cytoplasm there were numerous mitochondria and large quantities of rough endoplasmic reticulum (Figure 4B). Numerous droplets resembling lipid were observed in the basal portions of the cells, and this was confirmed by staining with Oil RedO (data not shown). The cells were attached to a well-developed basal lamina, beneath which were occasional stromal cell processes and collagen fibres.

**Figure 5:**
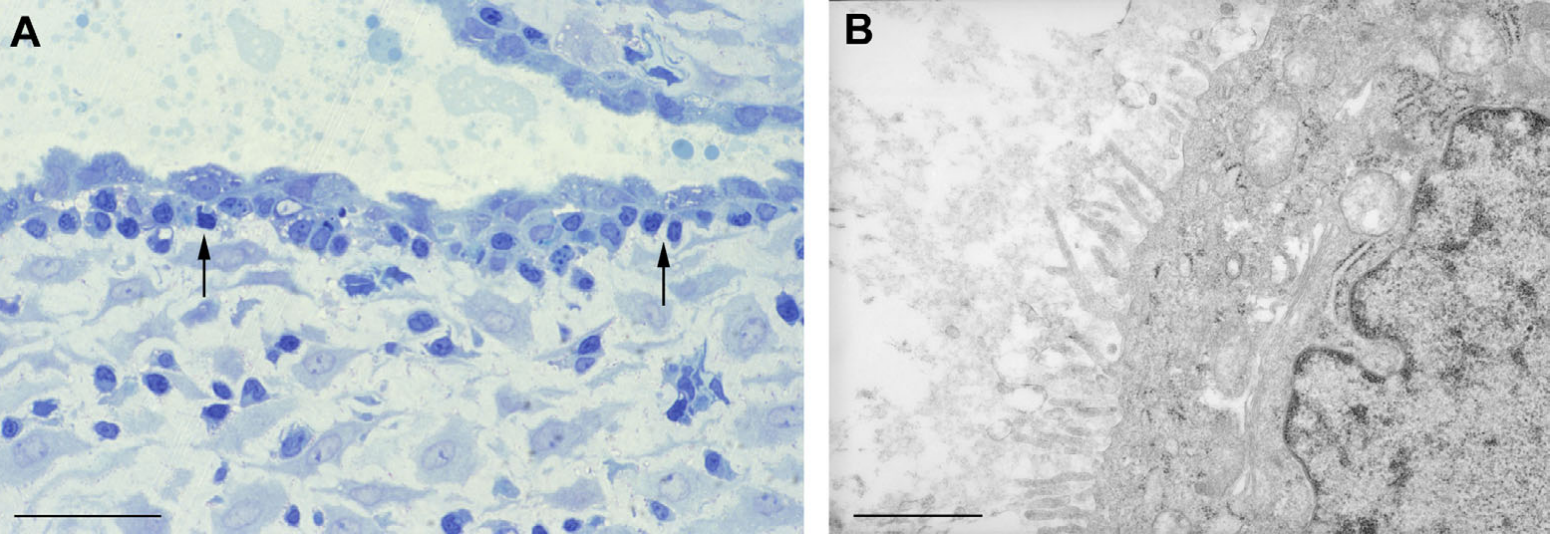
A) Photomicrograph of a 1 μm resin section of 10 week decidua. By now the epithelium is cuboidal in nature, although secretions are still present within the lumens. There appears to be an almost complete layer of additional cells (arrowed) beneath the basal lamina. Scale bar = 10 μm. B) At the ultrastructural level the cells appear more quiescent at this stage of gestation, although Golgi bodies and a few strands of rough endoplasmic reticulum remain. Scale bar = 1 μm.

**Figure 6:**
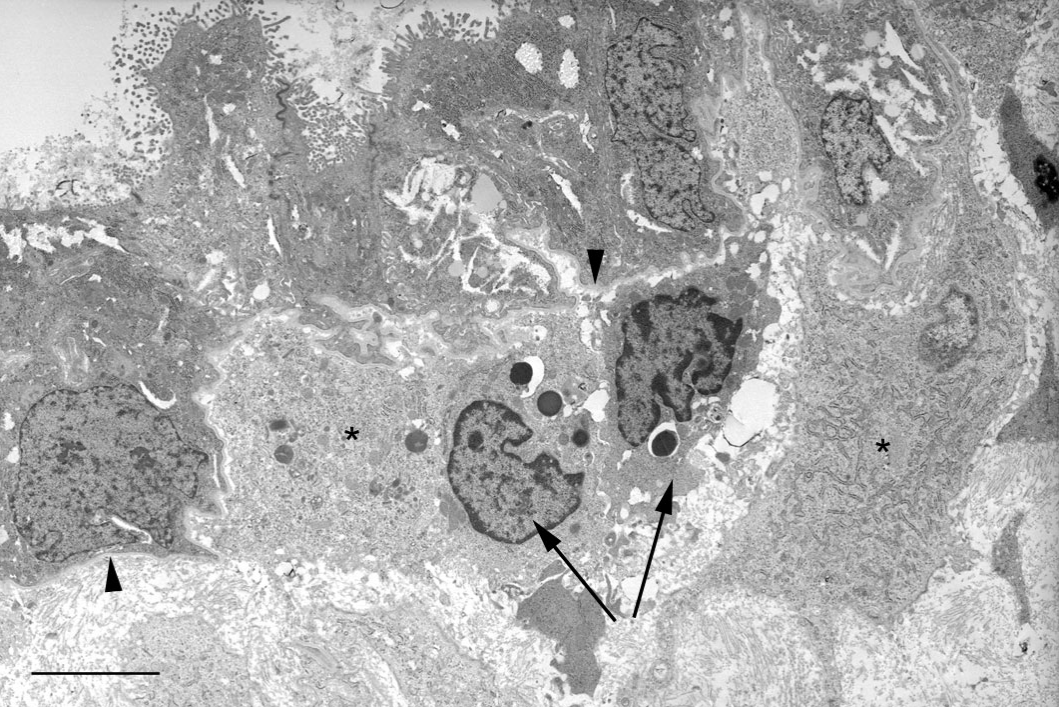
Low power transmission electron micrograph of 10 week decidua demonstrating the heterogenous population of cells accumulated immediately beneath the epithelial basal lamina (arrowheads) at this stage of gestation. The smaller cells (arrowed) with large numbers of granules resemble uterine NK cells, whereas the larger more electron lucent cells (asterisks) resemble decidual cells. Scale bar = 5 μm.

**Figure 7:**
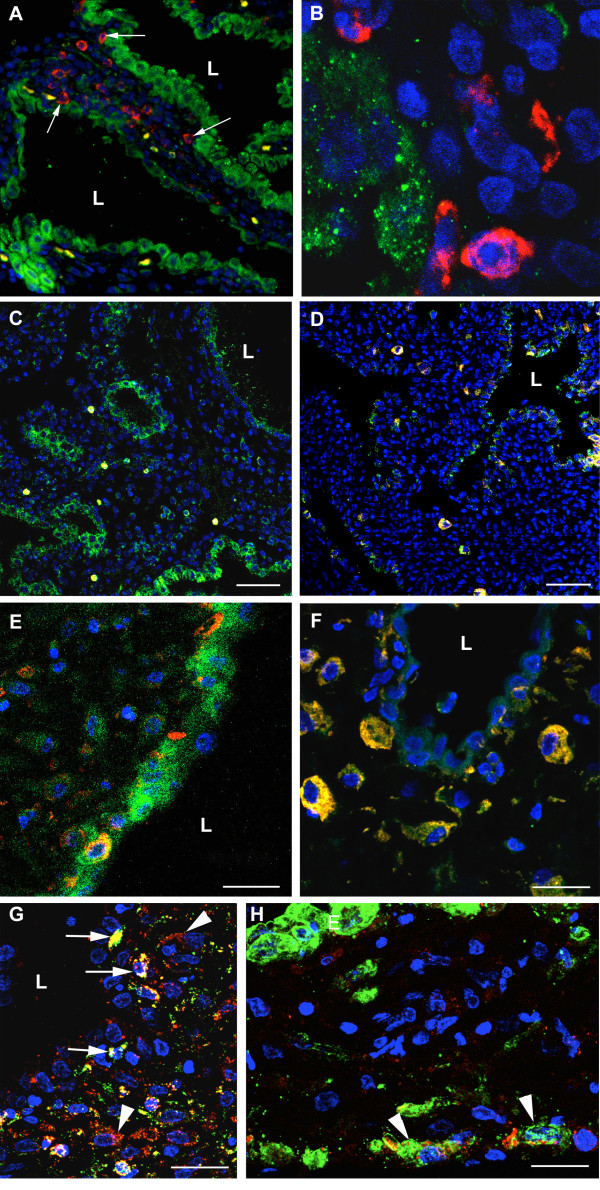
Confocal immunofluorescent images of decidua at 8 weeks (C, E, G, H) and 12 weeks (A, B, D, F) gestational age. In A) and B) the glandular epithelium has been immunolabelled for tocopherol transfer protein (green) and NK cells with CD56 (red). NK cells can be seen within the stroma between the glands, but also closely approximated (arrowed) to the basal lamina of the glandular epithelium. In C-F sections were immunolabelled for epidermal growth factor (EGF) (green) and CD56 (red). The epithelium reacts strongly at 8 weeks for EGF (C), but less so at 12 weeks (D). The NK cells lying beneath the glandular epithelium also react strongly for EGF (co-localisation yellow) (E and F). In G) and H) the sections were immunolabelled for human placental lactogen (red), and in G) for CD68 (green) and in H) for cytokeratin (green). Cells positive for both placental lactogen and CD68 (yellow) were considered to be macrophages, and were observed throughout the stroma but also closely approximated to the glandular epithelium (arrowed in G). Cells reacting only for placental lactogen, or for both placental lactogen and cytokeratin, were considered to be invading extravillous trophoblast cells (arrowheads in G and H), and were not found to be closely associated with the epithelium (E). Blue, DAPI; L, gland lumen. Scale bars C, D. = 60 μm and E - H = 30 μm.

By 10–11 weeks the cells were more cuboidal in nature with fewer apical projections (Figure 5A), although there was considerable variation between the glandular profiles even within the same sample. The apical cell membrane was frequently covered with long microvilli, and both Golgi apparatus and short strands of rough endoplasmic reticulum were present within the cytoplasm (Figure 5B). It was notable that other cell types were now present closely approximated to the deep surface of the basal lamina (Figures 5A and 6). One population possessed an irregularly shaped nucleus with dense peripheral heterochromatin, and osmiophilic membrane-bound granules were frequently present in the cytoplasm. Morphologically these resembled uterine natural killer (NK) cells, and this was confirmed using fluorescent immunohistochemistry and antibodies against CD56 (Figure 7F). The other cell type was larger, less osmiophilic and the cytoplasm resembled that of the stromal decidual cells. In order to attempt to identify these cells further immunostaining was performed for human placental lactogen and cytokeratin as markers for extravillous trophoblast, and CD68 as a marker for macrophages. Many invading trophoblasts and macrophages were present in the stroma between the glands, but only the latter were seen in particularly close proximity to the basal lamina (Figures 7G and 7H). The secretions within the glandular lumens reacted positively for placental lactogen, indicating communication with the intervillous space, as did the macrophages, suggesting phagocytic uptake of the hormone (Figure 7G).

**Figure 8:**
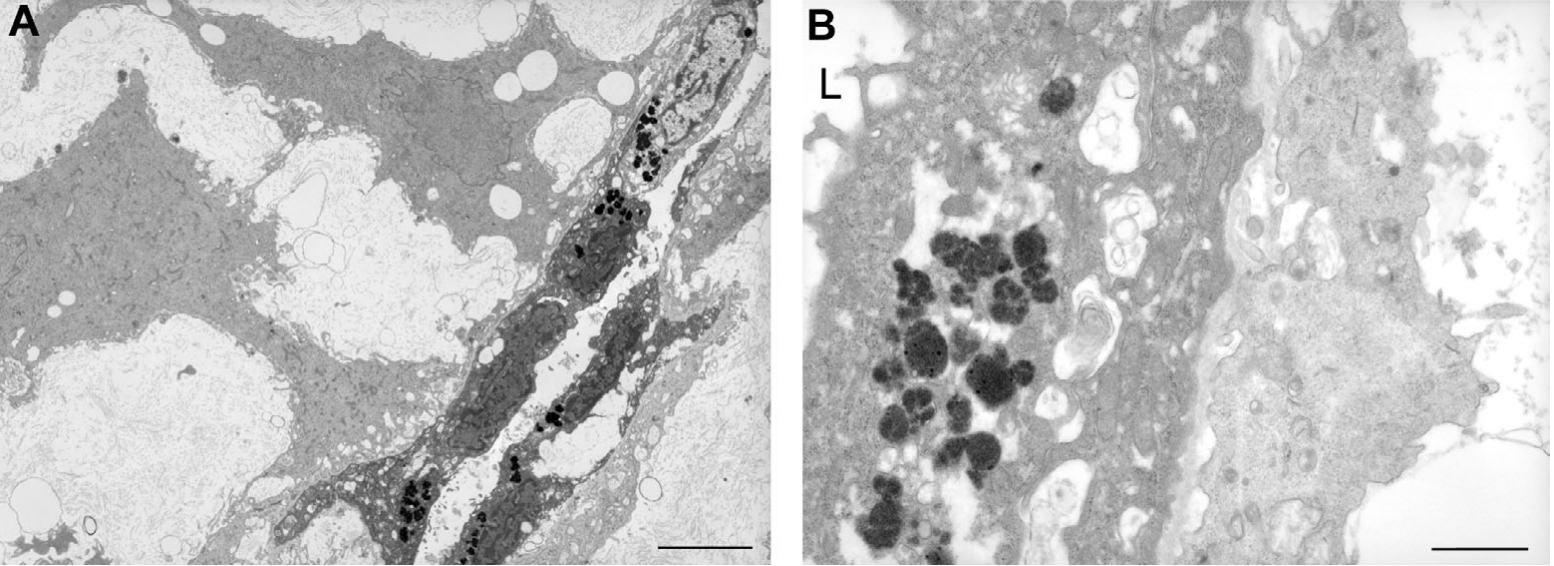
Transmission electron micrographs of 15 week decidua illustrating A) the flattened nature of the glandular cells at this stage of gestation, and B) the accumulation of a flocculent osmiophilic material in their cytoplasm. L, gland lumen. Scale bars = 5 μm and 1 μm.

By 14 weeks the glandular epithelial cells were markedly flattened and only a few short microvilli were present on the apical surface (Figure 8A). Few organelles were present in the cytoplasm, but instead there were numerous membrane vesicles containing a highly osmiophilic flocculent material resembling lipofuschin (Figure 8B). Decidual cells made extensive contact with the basal lamina beneath the epithelium, often extending long processes in order to do so (Figure 8A).

### Mucin and Cytokine production

**Figure 9:**
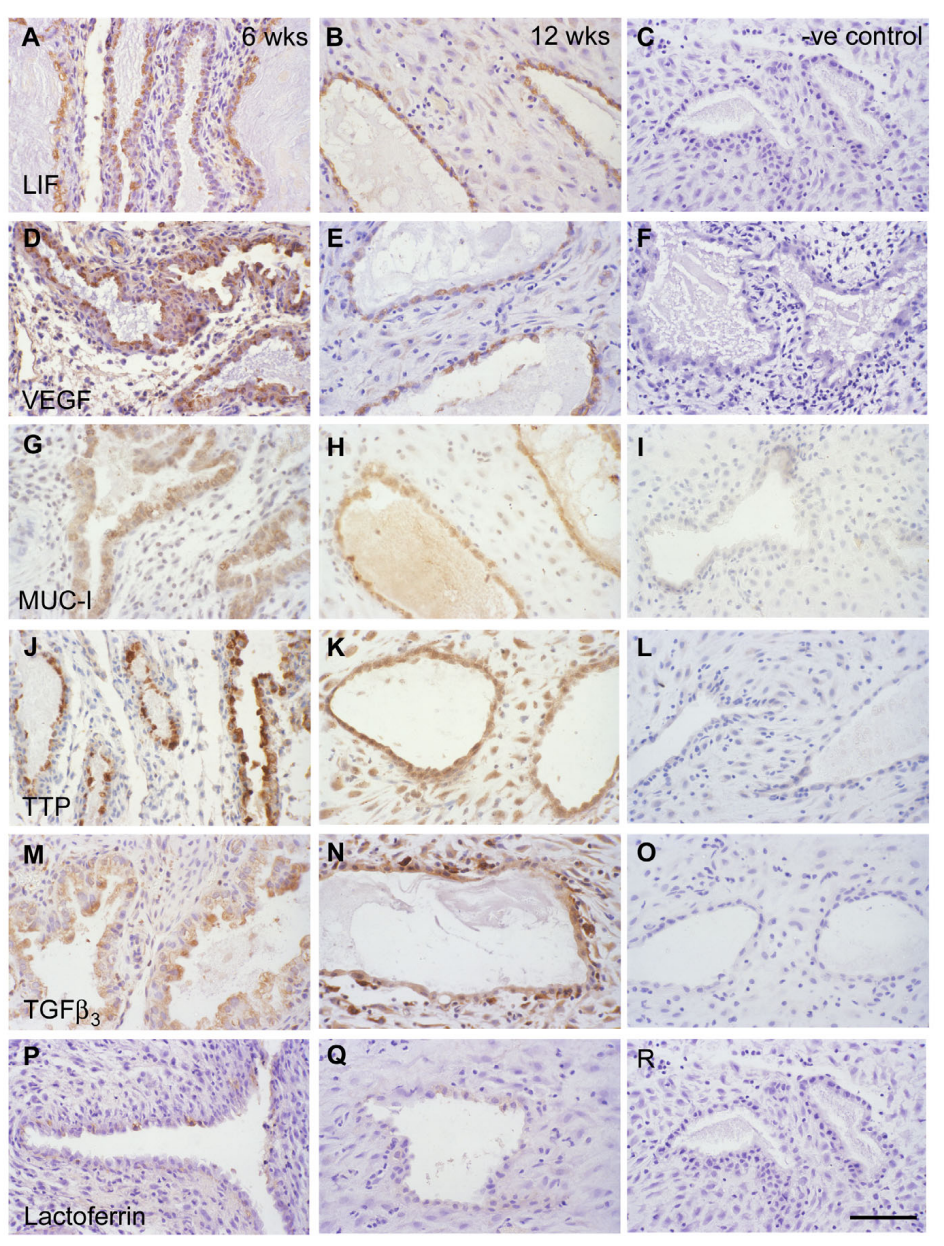
Photomicrographs of immunolabelled decidua at 6 weeks (A, D, G, J, M, P) and 12 weeks (B, E, H, K, N, Q) gestational age. The glandular epithelium reacted positively for LIF (A, B), VEGF (D, E), MUC-1 (G, H), alpha tocopherol transfer protein (J, K), TGFβ_3 _(M, N), and weakly for lactoferrin (P, Q). Negative controls; C, F, I, L, O and R. Scale bar = 200 μm.

The glandular epithelium reacted strongly for leukaemia inhibitory factor (LIF), VEGF and MUC-1 at all gestational ages from 5 to 14 weeks (Figure 9). In the early specimens the staining was particularly strong in the apical protrusions of the epithelial cells, whereas in the older cases it was more generalised throughout the cells. The secretions within the lumens also reacted positively for LIF and MUC-1.

A similar pattern was observed for alpha tocopherol transfer protein (TTP) and transforming growth factor beta (TGFβ_3_), although many of the decidual cells also reacted positively as gestational age increased (Figure 9). In addition, some of the interstitial cells and those just beneath the epithelium reacted intensely for TGFβ_3_. These were presumed to be macrophages and uterine NK cells.

The pattern was different for epidermal growth factor (EGF), for although the glandular epithelium initially displayed strong reactivity, the intensity reduced considerably by 9–10 weeks. By contrast, in the older specimens the cells immediately beneath the epithelium reacted strongly. As these cells also reacted positively for CD56 it was assumed they were NK cells (Figures 7C,7D,7E,7F).

Immunoreactivity for lactoferrin was weak even in the earliest specimens, although occasional cells reacted strongly. In the older specimens only faint staining could be identified (Figure 9).

In all cases the negative controls showed no staining.

### Fate of the secretions

**Figure 10:**
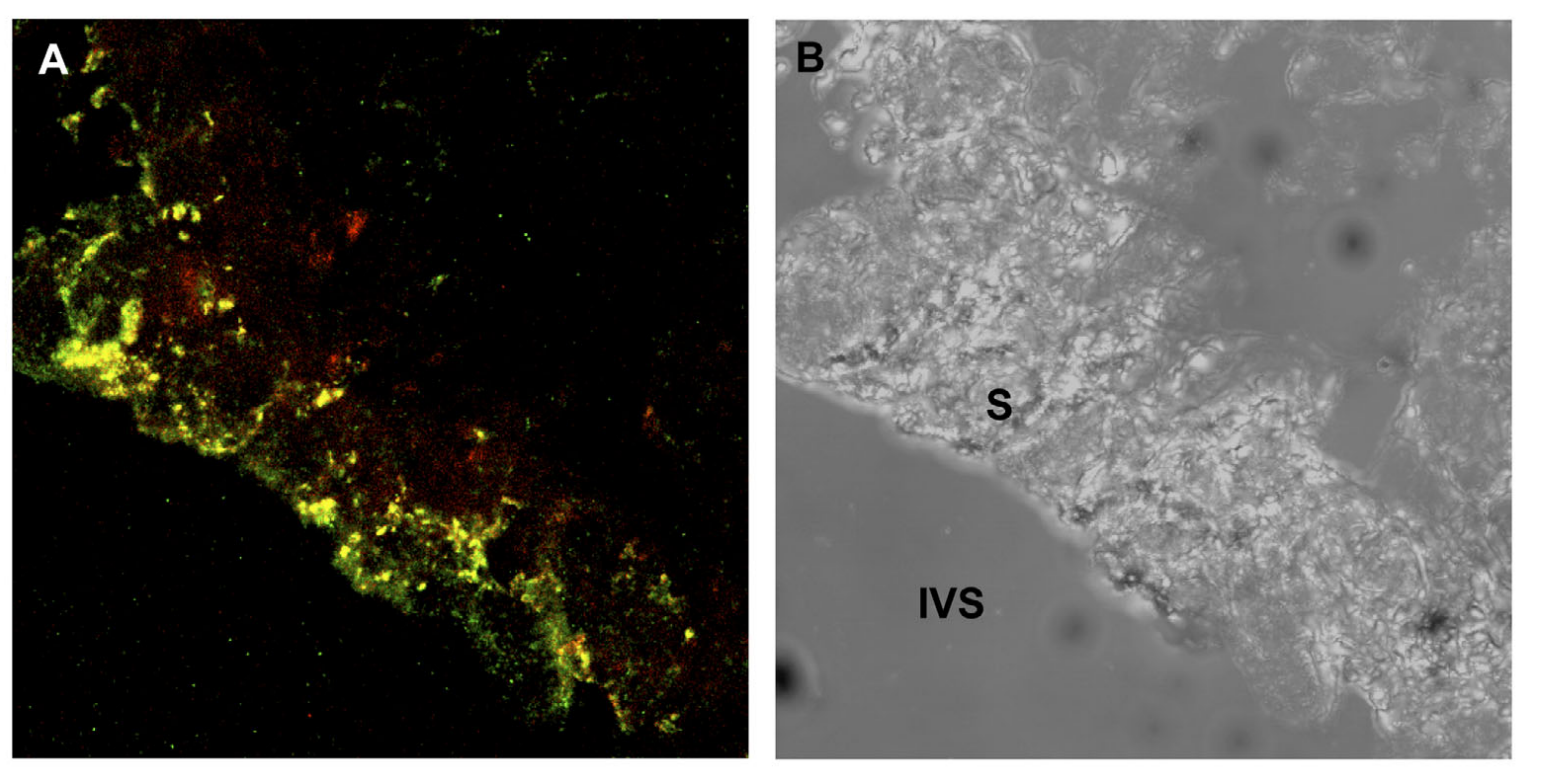
Confocal photomicrograph of a frozen section of an 8 week villus A) immunolabelled for glycodelin (green) and cathepsin D (red) and B) under phase contrast. Vesicles labelled solely for glycodelin predominate in the apical region of the syncytiotrophoblast (S), and those for cathepsin D in the basal region. In the mid-zone the two labels co-localise (yellow) indicating that maternal proteins enter the trophoblast digestion pathway. IVS, intervillous space.

In order to determine whether the glandular secretions taken up by the trophoblast enter the digestive pathway frozen sections of first trimester villi were dual-labelled for glycodelin, a glandular product, and cathepsin D, a marker of the lysosomal pathway. In the syncytiotrophoblast numerous vesicles immunoreactive exclusively for glycodelin were observed within the superficial layer of the syncytioplasm abutting the intervillous space, whereas lysosomes positive only for cathepsin D were observed in the basal region. In the midzone of the syncytioplasm the two labels were co-localised, indicating lysosomal fusion with the glycodelin-containing vesicles (Figure 10).

## Discussion

It is clear from this study of placenta-in-situ specimens that the uterine glands are still well-developed and highly active at 6 weeks of pregnancy, and that although there is considerable individual variation they gradually regress, both in terms of their length and the height of their epithelium, as the first trimester advances. Some of this variation may reflect differences in the thickness of the endometrium across the placental bed, for it was generally thinnest in the centre and thicker towards the periphery. Sampling at different sites may therefore yield different measurements. Nonetheless, by the start of the second trimester the endometrium beneath the placenta is very thin, the glandular epithelium is cuboidal and secretory organelles are no longer predominant. Indeed, the accumulations of osmiophilic material within the cytoplasm are reminiscent of lipofuschin, a characteristic of involuting or aging cells. These observations are consistent with a gradual shift from essentially histiotrophic nutrition of the human conceptus during the early first trimester to haemotrophic nutrition towards the start of the second trimester [3,20].

We previously reported that at 6 weeks gestational age the glandular epithelial cells closely resemble those during the luteal phase of the cycle, with large accumulations of glycogen within the apical portions of the cell [18,21]. In the normal menstrual cycle these accumulations disperse around days 23–24, but their persistence indicates that the corpus luteum of pregnancy maintains the glands in a highly active state during early gestation. The composition of the secretions from the uterine glands has been extensively investigated during the various phases of the menstrual cycle [22,23], but their contribution post-implantation has largely been ignored. The secretions are rich in carbohydrates, glycoproteins and, as demonstrated here, lipids. They therefore may provide an important source of nutrients for energy and elements for anabolic pathways within the feto-placental unit. The observation that glycodelin, a protein that is not expressed within placental tissues and so must be of maternal origin [24], enters the lysosomal digestive pathway within the syncytiotrophoblast supports this hypothesis. We have speculated that reliance on histiotroph during the period of organogenesis may protect the fetus from teratogenic damage by reactive oxygen species, for all mammalian embryos studied so far appear to rely heavily on anaerobic pathways during this period of development [25,26]. Once organogenesis is complete the oxygen concentration within the feto-placental unit rises as placental attachment and development occurs or, as in the case of the human, the maternal circulation to the placenta is fully established.

Besides acting as a source of nutrients our results also demonstrate that the glands express a wide variety of growth factors and cytokines, and so may play an important role in regulating placental development as in other species. Receptors for EGF have been localised immunohistochemically to the cytotrophoblast cells in the earliest stages of pregnancy, and on the syncytiotrophoblast in later gestation [27,28]. This switch parallels the dual actions of EGF reported, for in the earliest samples of 4–5 weeks EGF stimulated cytotrophoblast proliferation, whereas at 6–12 weeks it stimulated secretion of human chorionic gonadotropin (HCG) and placental lactogen [29]. Similarly, receptors for LIF have been demonstrated on first trimester villous and extravillous trophoblast populations, and on villous endothelial cells [30]. Addition of LIF to purified extravillous trophoblast cells had no effect on proliferation or integrin expression, but did inhibit forskolin-induced HCG production by BeWo cells in a dose-dependent fashion [30,31]. Receptors for VEGF have also been identified on the villous and extravillous trophoblast populations, and on villous endothelial cells [32,33], whilst TGFβ_3 _can modulate trophoblast differentiation between the proliferative and invasive phenotype [34]. Histiotroph may therefore potentially play significant roles in regulating trophoblast proliferation and differentiation during early pregnancy, as well as modulating placental vascularization.

Another group of proteins expressed by the glandular epithelium is that of transport carriers. TTP is a cytosolic protein first identified in the liver, but which has recently also been reported in the syncytiotrophoblast of the human placenta [35,36]. The high level of expression in the glandular epithelium suggests that histiotroph may be an important route for transfer of antioxidants during early pregnancy, increasing the defences of the feto-placental tissues against oxidative stress associated with onset of the maternal intraplacental circulation [3,37]. Lactoferrin is glycoprotein (molecular weight 82,400) traditionally associated with the transport of iron in breast milk. It was first identified immunohistochemically in the endometrial glandular epithelium and in their secretions, and although immunoreactivity was variable between specimens it was generally strongest during the late secretory phase of the cycle [38]. Here we identified significant staining only at the earliest gestational ages. The role of lactoferrin in the transport of iron is doubtful given the presence of transferrin receptors on the syncytiotrophoblast. Potentially, it may act as an antioxidant, for by forming stable complexes with free iron ions within the intervillous space it will reduce the possibility of generation of the highly toxic hydroxyl ion through the Fenton reaction [39,40]. It also possesses anti-microbial properties and so may contribute to the immune defences of the endometrium and early placenta [41].

Endometrial secretions may also modulate maternal immunological responses to the placental tissues. Thus glycodelin, which is released into the intervillous space, is immunosuppressive and functions as a direct T-cell inhibitor [9,42]. As gestation advances NK and stromal decidual cells migrate and come to lie closely approximated to the basal lamina of the glandular epithelium. The presence of NK cells within the glandular epithelium has been reported previously [43], and similar cells have been observed in an intraepithelial position in other species [44]. Whether the subepithelial cells we observed play a role in immune surveillance or support the epithelium in some other way is not clear at present, but the fact that they are immunopositive for EGF raises the possibility of paracrine signalling.

Because insufficient decidualization could have an impact on implantation and placentation, evaluation of the endometrial morphology by ultrasound has generated a lot of clinical interest. An endometrial thickness of 8 mm or more is considered to be favourable for implantation in humans [45], although this remains controversial as other authors have not found an association between endometrial thickness and pregnancy achievement [46]. One reason for this may be the fact that endometrial growth is not an homegeneous process, and that a single measurement of the endometrial thickness may not reflect the entire endometrial development. Within this context evaluation of the total endometrial volume with 3-D ultrasound could be a more accurate way of evaluating endometrial development [47]. Adequate endometrial thickness seems to be directly linked to uterine vascularization, and women with a good endometrial thickness on ultrasound but a poor intra-endometrial blood flow tend to have a poor reproductive outcome [48]. Uterine perfusion appears to regulate endometrial receptivity, and a high uterine resistance to blood flow is associated with recurrent miscarriages [49]. The visualisation of the endometrial circulation with 3-D doppler ultrasound appears to be an efficient parameter in predicting implantation in IVF cycles [50].

Attempts to correlate functional activity of the glands with pregnancy outcome have also met with mixed success. Thus, whilst reduced concentrations of MUC-1, LIF and glycodelin in uterine flushings have been reported in women suffering recurrent miscarriages [51,52], expression of these markers within the endometrium shows no significant association [53]. Why the glands should regress while maternal progesterone concentrations remain high is not known, but it would seem reasonable to assume that the decline of histiotrophic nutrition and the onset of haemotrophic exchange are co-ordinated in some way. How this might be achieved in the human is unknown at present.

## Authors' contributions

JH performed the tissue processing and colourimetric immunohistochemistry. TC-D performed the confocal immunofluoresence and dual-labelling. EJ performed the clinical procedures and collection of samples. GJB conceived the study and performed the electron microscopy and morphometric analysis.
